# Screening for a “trifecta” of executive function patterns in a large cohort of individuals with Parkinson’s disease

**DOI:** 10.1017/S1355617725101239

**Published:** 2025-08-18

**Authors:** Rachel N. Schade, Katie Rodriguez, Lauren E. Kenney, Adrianna M. Ratajska, Kelly D. Foote, Justin D. Hilliard, Michael S. Okun, Dawn Bowers

**Affiliations:** 1 Department of Clinical and Health Psychology, University of Florida, Gainesville, FL, USA; 2 Department of Neurology, Norman Fixel Institute for Neurological Diseases, University of Florida Health, Gainesville, FL, USA; 3 Department of Neurosurgery, Norman Fixel Institute for Neurological Diseases, University of Florida Health, Gainesville, FL, USA

**Keywords:** Neuropsychology, Parkinson’s disease, cognition, executive function, neuropsychological tests, memory

## Abstract

**Objective:**

This study examined three neurocognitive patterns or “clinical pearls” historically viewed as evidence for executive dysfunction in Parkinson disease (PD): 1) letter < category fluency; 2) word list < story delayed recall; 3) word list delayed recall < recognition. The association between intraindividual magnitudes of each neuropsychological pattern and individual performance on traditional executive function tests was examined.

**Methods:**

A clinical sample of 772 individuals with PD underwent neuropsychological testing including tests of verbal fluency, word list/story recall, recognition memory, and executive function. Raw scores were demographically normed (Heaton) and converted to z-scores for group-level analyses.

**Results:**

Letter fluency performance was worse than category fluency (*d* = −0.12), with 28% of participants showing a discrepancy of ≥ −1.0 SD. Delayed recall of a list was markedly poorer than story recall (d = −0.86), with 52% of the sample exhibiting ≥ −1.0 SD deficits. Lastly, delayed free recall was worse than recognition memory (d = −0.25), with 24% showing a discrepancy of ≥ −1.0 SD. These patterns did not consistently correlate with executive function scores. The word list < story recall pattern was more common in earlier than later PD stages and durations.

**Conclusion:**

Among the three pearls, the most pronounced was stronger memory performance on story recall than word lists, observed in more than half the sample. Only ¼ the participants exhibited all three neurocognitive patterns simultaneously. The variability in patterns across individuals highlights the heterogeneity of cognitive impairment in PD and suggests that intra-individual comparisons may offer a more nuanced insight into cognitive functioning.

## Introduction

Clinical decision-making in neuropsychological assessment often involves two complementary interpretive approaches. The first examines absolute impairment, typically through comparison to normative data, though this method has limitations related to individual variability and contextual factors. A more individualized approach assesses change relative to estimated or documented premorbid functioning, capturing meaningful decline that may not meet traditional impairment thresholds. The second approach focuses on intra-individual patterns of strengths and weaknesses across cognitive domains, aiding in the differentiation of global versus selective deficits. Together, these interpretive strategies enhance diagnostic precision and inform targeted intervention planning.

In this study, we examined whether individual differences in three specific neuropsychological patterns, commonly observed in Parkinson’s disease (PD), are present in a large PD sample and how these patterns relate to executive functioning. Executive dysfunction is one of the most well-recognized cognitive sequelae of PD, often emerging early due to dopamine depletion and degeneration within frontal-subcortical circuits, particularly the associative loop connecting the basal ganglia and prefrontal cortex (PFC) (Alexander et al., 1986; Brown et al., 2023; Hirano, 2021). The PFC itself consists of multiple subregions that may be differentially affected in PD, contributing to varied profiles of executive dysfunction (Foerde & Shohamy, 2011). Despite substantial functional overlap among these regions, differences in vulnerability likely underlie the considerable heterogeneity observed in executive dysfunction across individuals with PD (Arrigoni et al., 2024; Devignes et al., 2022; Kehagia et al., 2010). While traditional, standard executive function tests are used in neuropsychological assessments to gauge cognitive impairment in PD (e.g., Wisconsin Card Sorting Test, WCST) (Heaton & Staff, 1993), previous research has identified certain performance patterns that may indirectly reflect executive dysfunction in PD and offer additional interpretive value beyond traditional executive tasks.

One neuropsychological pattern researched in PD is the differential performance on letter versus category fluency tasks (Azuma et al., 2003; Pettit et al., 2013). Both types of verbal fluency have been localized to regions within the frontal and temporal lobes, but not equally: letter fluency is more dependent on frontal executive processes, whereas category fluency relies more heavily on temporal-lobe-based semantic retrieval (Troyer et al., 1998). This dissociation is supported by findings that frontal lobe damage disproportionately impairs letter fluency, while temporal lobe damage – as seen in Alzheimer’s disease – more strongly affects category fluency (Monsch et al., 1994; Rosser & Hodges, 1994; Troyer et al., 1998). Structural and functional neuroimaging support this distinction, with letter fluency activating primarily frontal regions and category fluency involving both frontal and temporo-parietal areas (Tupak et al., 2012; Vonk et al., 2018). In PD, even without dementia, frontal dysfunction is thought to underlie worse performance on letter fluency compared to category fluency (Azuma et al., 1997). However, findings have been mixed, with some studies reporting equivalent or even greater impairment in category fluency – often in older studies lacking cognitive stratification or normative comparison (Auriacombe et al., 1993; Azuma et al., 1997; Henry & Crawford, 2004).

A second observed cognitive pattern in PD is a performance discrepancy in delayed recall between word list and story memory tasks. Differences in these tasks are also thought to relate to frontal versus temporal localization of functions. Delayed recall of stories benefits from built in organization through semantically-related information, which is largely supported by temporal lobe structures (Helmstaedter et al., 2009; Lezak, 2004; Tremont et al., 2000). In contrast, word-list learning and recall rely more on self-generated organizational strategies of semantically related or unrelated words, which are more dependent on frontal lobe executive functions (Broadway et al., 2019; Kopelman & Stanhope, 1998; Tremont et al., 2000). As Cummings (1990) noted, individuals with subcortical dementias like PD are thought to perform better with structured information, whereas individuals with cortical dementias may not show a difference in performance with structured vs unstructured information recall. Accordingly, individuals with PD typically show greater impairment on word list recall due to executive dysfunction, while story recall remains relatively preserved (Helkala et al., 1989; O’Brien et al., 2009; Zahodne et al., 2011).

The third cognitive pattern concerns the difference between delayed free recall and recognition memory. Poor performance on both often suggests encoding or storage deficits, associated with a “cortical” memory profile. Conversely, impaired recall with preserved recognition has been viewed as a “retrieval deficit,” suggesting intact storage but difficulty accessing information without cues – a pattern often described in PD (Cummings, 1990). Squire and Wixted (2011) challenged this view however, noting that intact recognition does not equate to intact encoding or storage as recognition may rely on familiarity-based processes that demand less robust encoding than the recollection for recall. Thus, preserved recognition can occur with shallow encoding, complicating the attribution of poor recall solely to retrieval deficits. Indeed, studies have found both encoding deficits and impaired recognition in PD (Beatty et al., 2003; Hartikainen et al., 1993; Higginson et al., 2005; Tanner et al., 2015). Nonetheless, the more typical pattern in PD involves impaired delayed recall with relatively preserved recognition (E. Helkala et al., 1988), reflecting frontostriatal dysfunction affecting both encoding strategies and retrieval, rather than medial temporal lobe-related storage impairments (Carlesimo et al., 2022; Weintraub et al., 2004).

Although these three neuropsychological performance patterns have been frequently described in the literature, findings have been inconsistent - likely due to differences in methodologies and small sample sizes. Despite inconsistent empirical support, this “trifecta” of patterns has been described anecdotally in clinical practice and may be informally referenced as “clinical pearls” to support hypotheses of executive dysfunction. We clarify that our use of the term “trifecta” refers to commonly seen and practically useful patterns in clinical work with individuals with PD, not to formally established or universally accepted diagnostic criteria. The informal use of these patterns highlights the need for stronger empirical validation. Therefore, the primary aim of this study is to evaluate each of the three neuropsychological patterns in a large sample of individuals with PD. We also aimed to quantify the extent to which this “trifecta” of patterns co-occurred within individuals with PD. Specifically, we aimed to assess the percent of patients who exhibit these patterns overall, examine the degree of severity in the observed differences in test performance, and determine whether clinically meaningful differences are common within these neuropsychological patterns. Lastly, we explored the relationship between this “trifecta” and other executive function tests and other PD characteristics.

## Materials and methods

### Design

This study involved a retrospective chart review of individuals diagnosed with idiopathic PD who were seen at the University of Florida (UF) Health Norman Fixel Institute for Neurological Diseases. Study procedures were approved by the UF Institutional Review Board, with informed consent obtained in accordance with the Declaration of Helsinki and University and Federal standards. Data included demographic, clinical, and neuropsychological information.

### Participants

Participants included a clinical convenience sample of 772 individuals with idiopathic PD drawn from a large IRB-approved prospectively acquired clinical-research database (INFORM). Most participants were candidates for deep brain stimulation (DBS) surgery, indicating that motor symptoms were sufficiently bothersome and not well controlled by medication management. Inclusion criteria included: 1) a diagnosis of idiopathic PD made by a fellowship-trained movement disorders specialist and 2) neuropsychological evaluation between 2002 and 2022. Exclusion criteria included: 1) previous brain surgery (e.g., deep brain stimulation, pallidotomy); 2) history of epilepsy, stroke, brain injury, or other neurological diagnosis with ongoing cognitive sequela; and 3) evidence of significant cognitive impairment based on scores below 125 on the Dementia Rating Scale-2 (DRS-2; Johnson-Greene, 2004). Demographic and clinical data were obtained from the UF INFORM clinical-research database.

### Clinical measures

All participants underwent a comprehensive neuropsychological evaluation, including a cognitive screener and measures to assess functioning in multiple cognitive domains. The specific tests drawn from the full neuropsychological assessment for this study are highlighted in Table 1, as well as the raw scores used for analyses. Raw scores were converted to normed z-scores based on test-specific manuals or previously published norms (Heaton, 2004). Self-report mood and motivation scales were also included as part of a standardized neuropsychological battery as listed in Table 1.

All participants were “on” dopaminergic medication as part of routine clinical care. Most participants received PD-specific scales for disease staging (Hoehn & Yahr staging) and for gauging motor severity in response to dopamine medications (Unified Parkinson Disease Rating Scale-Part III) (Fahn & Elton, 1987; Goetz et al., 2004). UPDRS motor scores should be interpreted in the context of a pre-DBS cohort, in which many participants were referred due to suboptimal response to medication. As such, scores may not fully capture treatment efficacy.

### Statistical analyses

All analyses were conducted using SPSS Version 28 (IBM Corp, 2021). Data were examined for normality and outliers. Paired-sample t-tests were used to compare performance within each neuropsychological pearl, and we calculated the percentage of participants showing lower performance (z-difference <0) and clinically meaningful differences at z-score thresholds of ≤ −0.5, ≤ −1.0, and ≤ −1.5, representing increasing levels of severity in intra-individual performance discrepancies. This method, commonly used in the absence of anchor-based criteria, defines minimal clinically important difference as a change of ≥|0.5| SD and provides a standardized way to approximate meaningful intraindividual change. While clinical significance also depends on patient perceptions and score distributions, this approach offers a practical framework for interpretation.

We defined a meaningful difference of −1.0 standard deviation between tests, consistent with procedures for interpreting subtest discrepancies outlined in comprehensive assessments, including the Wechsler Adult Intelligence Scale–Fourth Edition. While comparison of separate tests is not the same as comparing subtests, this procedure aligns with evidence that such differences often exceed what is expected due to measurement error or normal variability, supporting their relevance for identifying significant cognitive strengths or weaknesses. For participants meeting the ≥ −1.0 SD threshold, we used independent t-tests and Chi-square tests to determine if there were demographic or clinical differences between those who did and did not show each neuropsychological pattern. FDR corrections (Benjamini & Hochberg, 1995) were applied to control for multiple comparisons in analyses comparing demographic and clinical variables between those who did and did not exhibit *a* ≥ 1.0 SD difference within each cognitive pattern.

Lastly, associations between neuropsychological pearls and executive function were explored via correlations and Chi-squared tests in a sub-sample of participants (*N* = 548) who had received three “executive” tasks assessing executive processes including cognitive inhibition (Stroop Color-Word (Golden, 1978), speeded set-shifting (Trail Making Test, Part B; (Reitan, 1992), and novel problem solving (Wisconsin Card Sorting Test-64 cards (Kongs et al., 2000), total errors). The z-score difference for each individual pearl was correlated with z-score performance on each classic task and an executive function z-score composite, as described in methods. Z-scores from each task were derived from test manuals and averaged to create a total executive function composite z-score. For each neuropsychological pattern, we correlated the z-score discrepancy (within-subject test pair difference) with individual executive task z-scores and a composite executive function z-score (calculated by averaging the three task specific z-scores). All correlation analyses were FDR corrected (Benjamini & Hochberg, 1995). The multiple correlational analyses between were FDR corrected. Exploratory Chi-squared analyses examined whether the prevalence of each neuropsychological pattern differed across PD clinical characteristics, including disease duration (categorized as early [≤ 5 years] vs. late [>5 years]) and Hoehn & Yahr disease stage.

## Results

### Sample characteristics

Demographic characteristics, scores on cognitive testing, and disease-related measures of the sample (*n* = 772) are depicted in Table 2. Overall, participants were largely non-Hispanic White (93.6%), male (72%), well-educated (15.2 ± 2.7), and in their mid-60s (65.0 ± 9.3). Most had tremor-dominant PD at the time of diagnosis with motor symptoms well-controlled with medication (Martínez-Martín et al., 2015). Motor severity, based on Hoehn and Yahr staging (subset, *n* = 540), ranged from stage 1 to 5 (mean = 2.35, SD = 0.62): Stage 1 (*n* = 13), 1.5 (*n* = 8), 2 (*n* = 289), 2.5 (*n* = 98), 3 (*n* = 108), 4 (*n* = 17), and 5 (*n* = 7). (Table 3)

### Clinical pearls

#### Pearl 1: letter fluency vs. Category fluency.

Letter fluency (z = −0.45 ± 1.1) was significantly lower than category fluency (z = −0.29 ± 1.2), though with small effect size (*t* (765) = −3.4, *p* = 0.001, Cohen’s *d* = −0.12). Among the 765 participants with both scores, 53% (*n* = 408) performed worse on letter than category fluency. Differences of ≤ −0.5 SD, ≤ −1.0 SD, and ≤ −1.5 SD were observed in 40% (*n* = 310), 28% (*n* = 212), and 16% (*n* = 120), respectively. Conversely, 44% (*n* = 333) performed better on letter fluency, with 28% (*n* = 214), 17% (*n* = 133), and 9% (*n* = 66) showing positive differences of ≥0.5 SD, ≥1.0 SD, and ≥1.5 SD, respectively.

Individuals who showed a ≤ −1.0 SD difference on Pearl 1 were younger at the time of testing (t (764) = 2.6, *p* = 0.01, *d* = 0.21) and had fewer years of education (t (764) = 3.2, *p* = 0.001, *d* = 0.26). No differences emerged in sex, race/ethnicity, DRS-2 scores, UPDRS motor scores, or self-reported mood symptoms after FDR-corrections (Table 4).

#### Pearl 2: delayed word list recall vs. Story recall.

Delayed recall of the Hopkins Verbal Learning Test-Revised (HVLT-R) word list (*z* = −0.93 ± 1.3) was significantly worse than delayed story recall from the WMS-III Logical Memory test (*z* = 0.15 ± 1.1), with a large effect size (*t* (752) = 23.7, *p* < 0.001, Cohen’s *d* = −0.86). Deficits of ≤ −0.5 SD, ≤ −1.0 SD, and ≤ −1.5 SD were found in 68% (*n* = 512), 52% (*n* = 388), and 34% (*n* = 254) of the 752 individuals with both scores, respectively. In contrast, 17% (*n* = 130) performed better on list recall, with differences of ≥0.5 SD (*n* = 79, 11%), ≥1.0 SD (*n* = 32, 4%), and ≥1.5 SD (*n* = 10, 1%).

Those with a ≤ −1.0 SD difference on Pearl 2 were more often male (χ^2^(1, *n* = 753) = 13.0, *p* < 0.001). No differences emerged in age, education, sex, race/ethnicity, DRS-2 scores, UPDRS motor scores, or self-reported mood symptoms after FDR-corrections (Table 4).

#### Pearl 3: delayed recall vs. Recognition discrimination.

Delayed recall performance on the HVLT-R (*z* = −0.93 ± 1.3) was significantly worse than recognition discrimination (calculated as the number of true positives minus false positives) on the yes-no recognition trial (*z* = −0.65 ± 1.2; *t* (724) = −6.7, *p* < 0.001) with small effect size (Cohen’s *d* = −0.25). Differences of ≤ −0.5 SD, ≤ −1.0 SD, and ≤ −1.5 SD were observed in 39% (*n* = 286), 24% (*n* = 172), and 14% (*n* = 105) of the sample respectively. Conversely, 40% (*n* = 291) performed better on delayed recall, with 21% (*n* = 151), 9% (*n* = 67), and 4% (*n* = 29) showing higher scores by ≥0.5, ≥1.0, and ≥1.5 SD, respectively.

Group-level analysis revealed that individuals with a ≤ −1.0 SD difference on Pearl 3 were significantly younger (*t* (723) = 2.8, *p* = 0.006; Cohen’s *d* = 0.26) compared to individuals who performed better than −1.0 SD (Table 4). There were no other group differences in demographics of PD clinical variables after FDR-corrections.

#### Co-occurrence of executive function trifecta.

**Figure 1.**
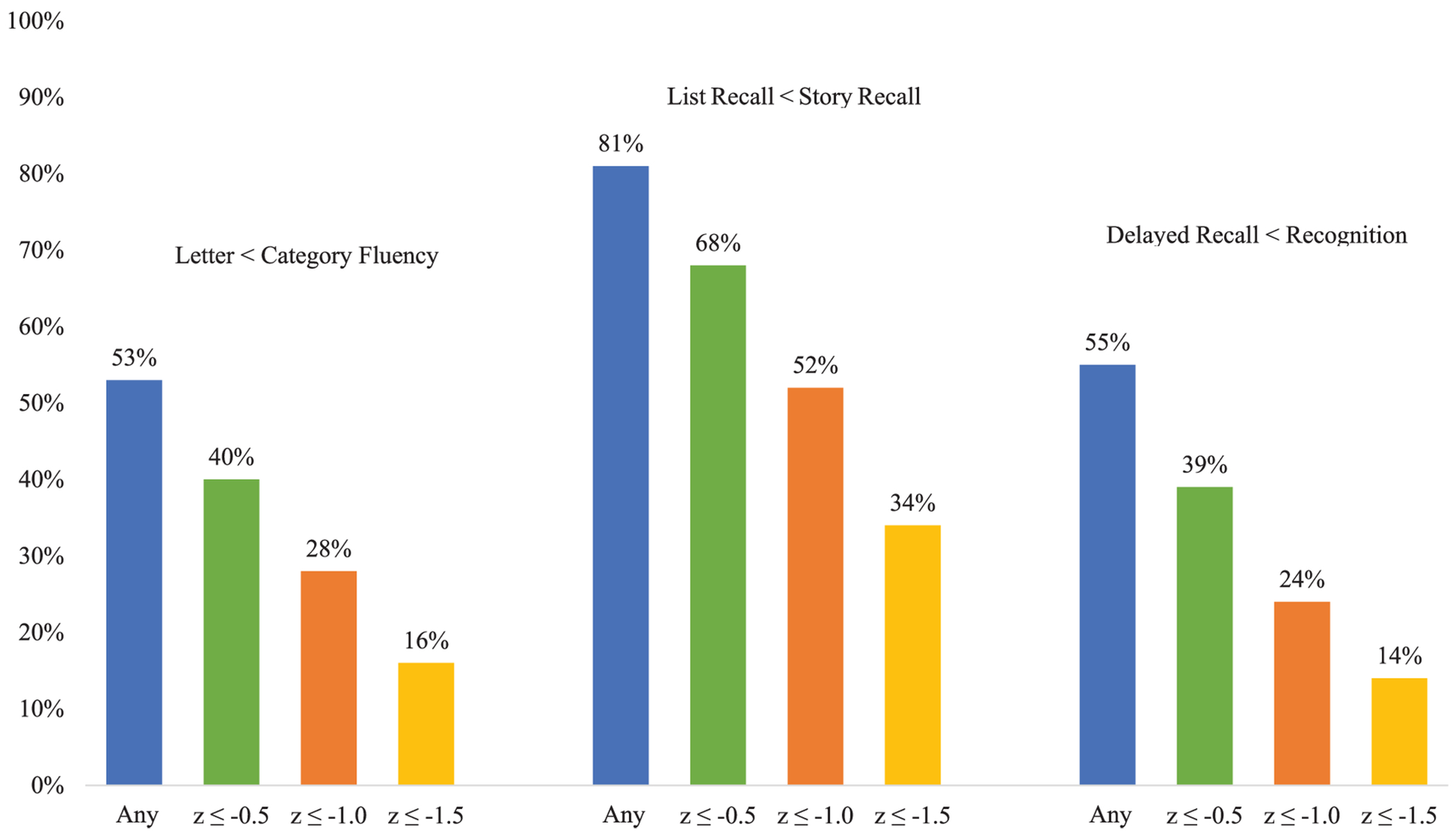
Cumulative percentage of the sample (n = 772) demonstrating z-score differences for each neuropsychological pearl.

**Figure 2.**
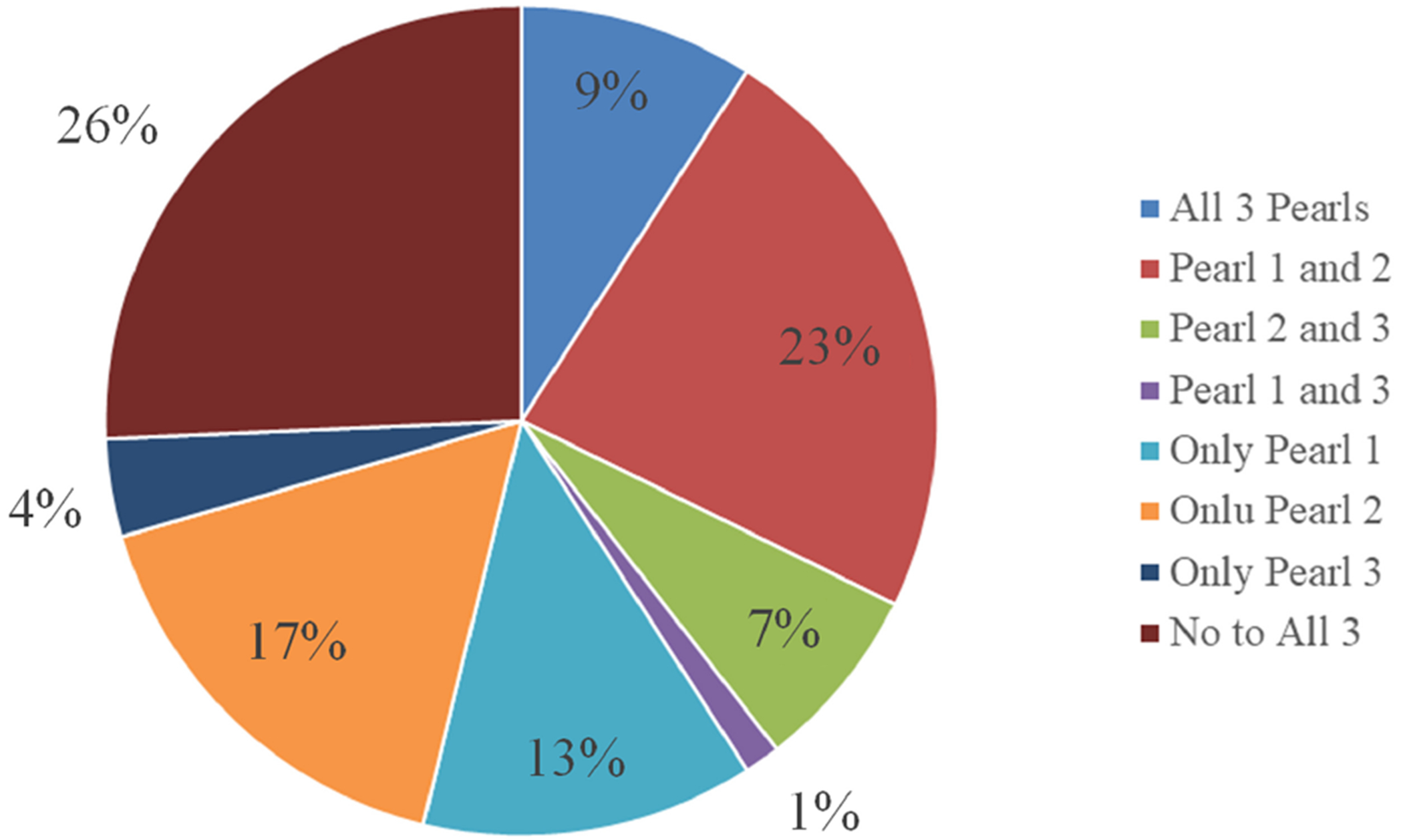
Frequency of co-occurrence of a trifecta of neuropsychological patterns (*n* = 716) with −1.0 SD z-score difference.

In addition to calculating the percentage of our sample displaying each neurocognitive pearl (Figure 1), we examined the co-occurrence of these three neuropsychological pearls within our sample (*n* = 716). For consistency, we used a z-score of difference of −1.0 between the two tests as a “yes” or “no” variable to indicate whether they did or did not demonstrate each cognitive pattern. Participants were grouped into all possible permutations, which came to 8 different groups, and we examined the frequency of the different groups (Figure 2). The largest percentage of the sample (28.3%, *n* = 203) did not demonstrate any of the three cognitive patterns. The second most frequent co-occurrence was for Pearl 1 and 2 (25.5%, *n* = 183), which appeared to be driven largely by Pearl 2 as 14.2% (*n* = 102) of this sample demonstrated this pattern only.

#### Comparison to classic executive function tests.

A subset of 548 participants completed three executive function tests, the Stroop Color-Word test (average *z* = −0.34 ± 1.0, range = −3.00 to 3.00), Trail Making Test Part B (TMT-B; average *z* = −0.95 ± 1.4, range = −3.00 to 3.00), and Wisconsin Card Sorting Test (WCST) total errors (average *z* = −0.58 ± 1.2, range = −3.10 to 2.50) (Table 3). The distribution of scores across each of these tasks reflects a range from impaired to strong performances. To explore the relationship between performance on these executive function tests and the presence of neuropsychological pearls, we first conducted FDR-corrected correlations between the z-score differences within each pearl, z-score performance on each classic task, and the executive function z-score composite, as described in methods. Only Pearl 3 demonstrated a significant, albeit small, correlation with executive function composite scores (*r* = 0.132, *p* = 0.003), suggesting limited overlap.

With this sample subset, we then categorized participants into three groups based on their executive function composite scores: a “low” executive function group (z ≤ −1.0) (*n* = 148), a “within normal limits” (WNL) group (−1.0<z<1.0) (*n* = 376), anda “high” executive function group (z ≥1.0) (*n* = 24). Chi-square tests revealed no significant differences in the proportion of individuals displaying *a* ≥1.0 SD discrepancy in any of the three pearls among the low, WNL, and high executive groups. These findings suggest that traditional executive tasks did not predict the neuropsychological patterns observed in this sample.

#### Relationship of PD Variables to Executive Function Trifecta.

Given that executive dysfunction often emerges early in PD, we explored whether disease duration or severity was associated with the presence of neuropsychological pearls. Disease duration (years since symptom onset) was unrelated to the executive function composite and each neuropsychological pearl’s z-score difference, suggesting that longer disease duration was not associated with greater likelihood of these patterns. When participants were divided into two groups based on disease duration: “early” (≤5 years) and “late” (>5 years), chi-square analyses revealed that a greater percentage of individuals in the early group exhibited Pearl 2 compared to the late group (X^2^(1, *n* = 741) = 4.0, *p* = 0.044).

We also examined pearl prevalence across stages of disease severity using Hoehn & Yahr (H&Y) ratings by dividing participants into “early-stage” (H&Y 1–2) and “mid-stage” (H&Y 2.5–3). Individuals in H&Y stages 4–5 (*N* = 23) were excluded due to small sample size. Chi-squared tests revealed more early-stage participants demonstrated Pearl 2 compared to midstage participants (X^2^ (1, *n* = 506) = 3.9, *p* = 0.049). Together, these results suggest that worse delayed recall of unstructured information compared to structured information is more common in the earlier stages and durations of PD. This may reflect the early impact of executive dysfunction, which may later be overshadowed by broader impairments in memory or other cognitive domains as the disease progresses.

## Discussion

This study sought to learn whether a “trifecta” of previously identified neuropsychological patterns could be validated in a large cohort of individuals with Parkinson’s disease. The focus was three key cognitive pearls: letter fluency < category fluency, delayed recall of a word list < story recall, and delayed recall < recognition. Statistically, our results supported these hypothesized cognitive patterns. On average, individuals with PD performed statistically worse on letter fluency, delayed recall of a word list, and delayed recall compared to recognition.

When going beyond statistical significance of average scores, our results revealed that the “trifecta” of neuropsychological patterns or “clinical pearls” were not uniformly present, and many did not show clinically meaningful differences between tests within a given neuropsychological pattern (Figure 1, Figure 2). Indeed, only Pearl 2 (word list vs. story memory) showed a large effect size with at least half the sample showing a clinically meaningful difference (−1SD) in performance. The other two patterns yielded small effect sizes, and only a minority exhibited differences of one standard deviation or greater. This variability suggests that while certain cognitive patterns may emerge at the group level, individual differences in cognitive decline and neuropsychological profiles in Parkinson’s disease are substantial, underscoring the need for individualized assessment.

We further explored how these patterns related to broader executive functioning (Stroop Color-Word, TMT B, WCST). There was marked individual variability in performance across these executive function tasks, in line with recent reports about differing rates of cognitive decline (Fereshtehnejad et al., 2025). Pearls were either weakly or unrelated to performance on classic executive function tests. This discrepancy may reflect differences in task-specific demands, the multifaceted nature of executive function (i.e., different neural substrates involved in different tasks), and the varied use of compensatory strategies or cognitive reserve. Furthermore, stratifying participants by executive function status revealed no significant differences in the prevalence of any pearl, except Pearl 3 (recall < recognition), which was significantly correlated with executive performance. Notably, Pearl 2 (word list vs story) was more prevalent in individuals with shorter disease duration and lower Hoehn & Yahr stage, suggesting it may be a more prominent early disease marker that diminishes with broader cognitive decline as more diffuse or non-frontal cognitive deficits arise.

Our findings, based on what is perhaps the largest sample of individuals with PD to date, align with the broader literature in emphasizing the complex and heterogeneous nature of cognitive impairments in PD. Prior studies have yielded mixed results, with both confirmative and contradictory findings regarding neuropsychological patterns, particularly in verbal fluency. For example, there are at least eight studies that found worse performance on letter vs category fluency (Barbosa et al., 2017; Bayles et al., 1993; Gabrieli et al., 1996; Galtier et al., 2017; Jaywant et al., 2014; Monsch et al., 1994; Rosser & Hodges, 1994; Suhr & Jones, 1998; Troyer et al., 1998). In contrast, over ten other studies have found the opposite, with better performance on category compared to letter fluency (Auriacombe et al., 1993; Beatty et al., 1989; Koerts et al., 2013; Matison et al., 1982; Raskin et al., 1992) or no difference between the two types of fluency at all (Azuma et al., 1997; Dadgar et al., 2013; Flowers et al., 1995; Gurd & Ward, 1989; Hanlly et al., 1990; McDowd et al., 2011; Obeso et al., 2011; Piatt et al., 1999; Troyer et al., 1998). Notably, a meta-analysis of verbal fluency performance in PD showed more impairment on category fluency than letter, though both were found to be related to psychomotor speed more than executive dysfunction (Henry & Crawford, 2004), which has been corroborated in at least two other studies (Koerts et al., 2013; McDowd et al., 2011). Some of the aforementioned studies also did not directly compare letter vs category fluency performance within a group, only noting that both verbal fluencies were impaired compared to controls (Azuma et al., 1997; Dadgar et al., 2013; Gurd & Ward, 1989; Obeso et al., 2011; Troyer et al., 1998). Methodological differences, such as the number of fluency trials, the specific letter or categories tested, and the norming standards use, may account for inconsistent findings across studies. In the current study, participants on average performed worse on three letter fluency trials compared to a single trial of category fluency, which raises multiple possible considerations. One possibility is that the cumulative demand of multiple trials, especially on a task potentially more reliant on executive control, may reveal subtle impairments that a single trial cannot capture. Alternatively, poorer performance across three trials may reflect fatigue or difficulty maintaining verbal output under increasing cognitive load. has shown that in healthy older adults, category fluency typically declines more with age than letter fluency (Gladsjo et al., 1999) suggesting that the opposite pattern found in our sample is unlikely to be explained by normative aging effects alone. Moreover, both letter and category fluency tasks were conormed and standardized using the revised Heaton norms, allowing for a direct, demographically adjusted comparison in performance.

At least three studies have demonstrated worse word list delayed memory compared to spared delayed recall of stories in PD (Hartikainen et al., 1993; Lafo et al., 2015; Zahodne et al., 2011) and in individuals with “significant” executive dysfunction (Tremont et al., 2000). A similar study found worse performance on short delay recall of a word list in a group “with executive dysfunction” compared to the group without executive dysfunction, but no difference in long delay (Brooks et al., 2006). One PD study did not find a difference in delayed recall performance between list and stories using the Repeatable Battery for the Assessment of Neuropsychological Status (RBANS), but statistical analyses were only conducted using a difference score, rather than directly comparing list and story performances (Beatty et al., 2003). These mixed findings may in part reflect differences in test sensitivity and normative frameworks across studies. For instance, the California Verbal Learning Test (CVLT) includes more trials, structured semantic categories, and greater demands on learning and retrieval compared to other word list tasks like the HVLT, potentially making it more sensitive to executive dysfunction. Differences in demographic norming between the HVLT-R and the WMS-III Logical Memory may have also influenced performance classification. However, the study by Zahodne and colleagues demonstrated the same pattern (poorer performance on a word list versus stories) when both memory tasks were co-normed together from the same reference group (i.e., Weschler Memory). Nevertheless, discrepancies in memory performance patterns may be shaped not only by underlying cognitive deficits but also by the psychometric properties and normative context of the measures used.

Lastly, at least five studies have provided evidence supporting the third clinical pearl, namely impaired verbal delayed recall with relatively spared recognition (Auriacombe et al., 1993; Brooks et al., 2006; Taylor et al., 1986, 1990). Conversely, six studies found similar impairments in delayed recall and recognition compared to controls suggesting additional dysfunction in encoding (Beatty et al., 2003; Brønnick et al., 2011; Hartikainen et al., 1993; Higginson et al., 2005; Tanner et al., 2015). However, most of these studies did not directly compare performances in the PD cohort alone (Beatty et al., 2003; Brønnick et al., 2011; Hartikainen et al., 1993; Higginson et al., 2005; Tanner et al., 2015). Further support for encoding and retrieval difficulties comes from a meta-analysis showing impaired delayed recall and recognition in PD, even in the absence of dementia, though with low effect sizes (Whittington et al., 2000). Importantly, Squire and colleagues have cautioned against interpreting preserved recognition as definitive evidence of intact encoding or storage, as recognition memory can often rely on familiarity-based processes that do not require deep or elaborative encoding (Squire & Wixted, 2011). Thus, relatively intact recognition can occur even when encoding is shallow, complicating the inference that poor recall reflects a pure retrieval deficit. Moreover, effective retrieval of episodic information is thought to reactivate encoding engrams, underscoring the interdependence of these processes and the challenges in using neuropsychological tests to disentangle them cleanly (Alvarez & Squire, 1994; Nyberg et al., 2000; Squire & Kandel, 2000).

Overall, the conflicting research findings are likely a result of the interplay of methodological and sample-related factors. Possible methodological differences include the smaller sample sizes, determination of dementia based on varying criteria, use of different neuropsychological tests and process scores to assess memory and verbal learning, and use of varied statistical methods. Sample characteristics may also account for discrepancies observed. Smaller studies, particularly earlier studies, may be constrained by a narrower range of demographic (e.g., age) or PD characteristics (e.g., motor severity, disease duration) and likely lack the statistical power to capture the full diversity of cognitive profiles. In our study, participants’ age ranged from 30–90, which is an exceptionally large age range compared to many studies that focus on older adults with PD. It is believed that young onset PD individuals tend to have less severe motor progression and cognitive impact compared to late onset PD (Diederich et al., 2003; Pagano et al., 2016; Santos-García et al., 2023). As a result, the inclusion of younger individuals in our sample may have diluted the overall prevalence or severity of neuropsychological differences typically observed in older PD cohorts. Additionally, our sample demonstrated relatively mild motor severity and a wide range of executive dysfunction overall, which may also explain the lower frequency of robust or clinically meaningful differences in performance across the patterns we examined. It is also likely that at least some individuals have co-occurring neuropathology like Alzheimer’s disease or limbic-predominant age-related TDP-43 encephalopathy, which could also be impacting the presenting neurocognitive profiles (Fan et al., 2021; Nelson et al., 2019). Lastly, few studies have examined clinically meaningful differences in neuropsychological patterns to determine the percentage of individuals who display a certain pattern. Our findings suggest that the broader age range and larger sample size employed here allowed for a potentially more comprehensive representation of cognitive variability.

This study has several limitations. First, the use of a convenience sample – primarily individuals undergoing neuropsychological evaluations for DBS candidacy – introduces selection bias. These patients often represent a specific subset of PD (e.g., tremor dominant, less cognitively impaired), limiting generalizability. Second, we lacked data on cognitive diagnoses (e.g., amnestic vs. non-amnestic MCI), which precluded analyses by MCI subtype. Third, we used test-specific normative data consistent with clinical practice, which introduced variation in comparison groups – particularly affecting Pearl 2. The sample was also predominantly non-Hispanic White, well-educated individuals, reducing. This not only significantly reduces applicability of these findings to more diverse sociocultural populations, but future studies in more diverse groups would be potentially limited by the specific normative groups themselves, as these lack consideration of individuals from diverse backgrounds and sociocultural factors that can influence cognitive performance in meaningful ways (Byrd & Rivera-Mindt, 2022). Additionally, all PD participants were assessed while “on” their standard dopaminergic medications, but their medication usage was not formally tracked during the 2–3-hour evaluation. Variability in medication levels, including potential “wearing off” effects or concerns with excessive dopamine (e.g., overdose hypothesis), may have influenced cognitive performance and should be considered when interpreting the results. Lastly, there is limited research on direct comparisons of tests and common neurocognitive patterns in healthy, cognitively intact older adults. Future studies should prioritize exploring typical patterns of relative strengths and weaknesses in aging to better contextualize findings in PD and other neurodegenerative conditions.

Our findings suggest that incorporating pattern-based interpretation – focusing on within-person variability and relative cognitive strengths and weaknesses – may provide more nuanced insights into the cognitive changes associated with Parkinson’s disease. However, given the lack of robust findings, this study underscores the importance of critically evaluating commonly cited neuropsychological patterns of relative impairment – especially those derived from small or older studies. The presence or absence of these patterns alone should not be viewed as a definitive indicator of cognitive status or diagnosis. Instead, when assessing executive dysfunction in PD, such patterns should be interpreted within the broader context of a comprehensive neuropsychological evaluation.

## Figures and Tables

**Table 1.** Neuropsychological tests and self-report measures within each cognitive domain composite

Cognitive domain	Tests	Raw score used in study
**Global cognitive screener**	Dementia Rating Scale-2 (DRS-2)	Total Correct
**Verbal recent memory**	Hopkins Verbal Learning Test- Revised (HVLT-R) WMS-III Logical Memory	Delayed Total Recall, Recognition Discrimination Delayed Total Recall
**Executive function**	COWA Letter Fluency (FAS) Stroop Test, Color-Word Trial Trail Making Test- Part B Wisconsin Card Sorting Test	Total Number of Words (all 3 trials) Total number of correct items Completion time Total Number of Errors
**Language**	Category Fluency (Animals)	Total Number of Words
**Mood/Motivation**	Beck Depression Inventory-II (BDI-II) State Trait Anxiety Inventory Apathy Scale	Total Score Percentile Trait Anxiety Total Score

*Note:* WMS-III = Wechsler Memory Scale-Version III (Wechsler, 1997); HVLT-R = Hopkin’s Verbal Learning Test-Revised (Brandt, 1991); Letter Fluency (FAS) (Tombaugh et al., 1999); Stroop Test is the Golden version (Golden, 1978); TMT-B = Trail Making Test Part B (Reitan, 1992); Wisconsin Carding Sorting Test-64 (hand administration) (Kongs et al., 2000); Category Fluency (Animals) (Tombaugh et al., 1999); BDI-II (Beck et al., 1996; Leentjens et al., 2000), AS (Leentjens et al., 2008; Starkstein et al., 1992), STAI (Knight et al., 1983; Spielberger, 1983).

**Table 2.** Sample demographic, clinical, and cognitive (z-score) characteristics

Measure	Overall Sample (*n* = 772) except where indicated		

Variable	Mean/%	SD	Range
Age (years)	65.0	9.3	30-90
Education (years)	15.2	2.7	6-22
% Male	72.0%	–	–
% White non-hispanic	93.6%	–	–
Disease duration (years)	9.59	5.3	0.5-30
UPDRS III, on medication	26.0	11.0	1-74
Hoehn & Yahr staging (n = 540)	2.36	0.62	1-5
PD motor subtype	75.7%	–	–
Tremor predominant	21.1%	–	–
Akinetic-rigid	3.1%	–	–
PIGD	136.5	4.7	125-144
Dementia rating scale-2, raw total	10.44	7.8	0-54
BDI-II	11.7	6.4	0-30
Apathy scale	58.8	31.0	3-100%ile
%ile Trait anxiety (STAI)	–	–	–
HVLT-R	−0.87	1.02	−3.90 - 2.40
Trial 1	−0.93	1.28	−4.00 - 1.40
Delay	−0.65	1.23	−3.00 - 1.40
Recognition	0.14	1.09	−3.00 - 3.00
WMS-III logical memory	−0.45	1.09	−3.30 - 2.50
Delay	−0.30	1.12	−4.00 - 3.80
Letter fluency (total; FAS)			
Category fluency (animals)			

*Note:* UPDRS III = Unified Parkinson’s Disease Rating Scale motor scale, BDI-II = Beck Depression Inventory-II, STAI = State Trait Anxiety Inventory, WMS-III = Wechsler Memory Scale-Version III (Wechsler, 1997); HVLT-R = Hopkin’s Verbal Learning Test-Revised (Brandt, 1991); Letter Fluency (FAS) (Tombaugh et al., 1999); Category Fluency (Animals) (Tombaugh et al., 1999); BDI-II (Beck et al., 1996; Leentjens et al., 2000), AS (Leentjens et al., 2008; Starkstein et al., 1992), STAI (Knight et al., 1983; Spielberger, 1983). Cognitive tests scores provided are z-scores as described in methods.

**Table 3.** Sample average performance on executive measures (z-scores)

Measure	Overall Sample (*n* = 500)		

	Mean	SD	Range
Stroop test, color-word trial	−0.34	1.04	−3.00 - 3.00
Trail making test- part B	−0.95	1.38	−3.00 - 3.00
Wisconsin card sorting test, total errors	−0.58	1.17	−3.10 - 2.50

*Note*: Stroop Test is the Golden version (Golden, 1978); Trail Making Test Part B (Reitan, 1992); Wisconsin Carding Sorting Test-64 (hand administration) (Kongs et al., 2000).

**Table 4.** Descriptive characteristics between individuals with and without at least a −1.0 standard deviation difference in performance on pearls 1-3

	Age	Education (years)	% Male	% White non-hispanic	Disease duration (years)	UPDRS III, on medication	DRS-2 total score	BDI-II	Apathy scale	Trait anxiety (%ile)
Pearl 1										
Z ≥ −1.0 SD (*N* = 212)	63.7 (9.8)*	14.6 (2.7)*	71.10%	94.90%	10.5 (12.5)	24.5 (10.3)	136.3 (4.4)	10.4 (7.4)	11.5 (6.3)	59.4 (30.0)
Z < −1.0 SD (*N* = 554)	65.6 (8.9)	15.3 (2.7)	73.60%	91.50%	9.9 (10.9)	26.8 (11.2)	136.6 (4.8)	10.5 (8.0)	11.8 (6.4)	58.5 (31.5)
Pearl 2										
Z ≥ −1.0 SD (*N* = 388)	65.1 (8.7)	15.1 (2.8)	77.60%*	92.20%	9.8 (11.6)	25.7 (11.2)	136.2 (4.7)	10.2 (7.3)	11.5 (6.3)	58.0 (30.7)
Z < −1.0 SD (*N* = 365)	64.9 (9.8)	15.2 (2.7)	65.80%	96.20%	10.2 (10.6)	26.6 (10.8)	137.0 (4.6)	10.5 (8.0)	11.8 (6.4)	59.1 (31.3)
Pearl 3										
Z ≥ −1.0 SD (*N* = 144)	63.0 (9.1)*	14.8 (3.0)	73.60%	94.70%	8.3 (5.0)	26.3 (11.0)	136.3 (4.1)	10.6 (7.9)	12.2 (6.6)	59.4 (30.5)
Z < −1.0 SD (*N* = 581)	65.4 (9.3)	15.2 (2.7)	71.10%	91.60%	8.8 (7.7)	26.1 (11.1)	136.7 (4.8)	10.6 (47.9)	11.5 (6.4)	58.0 (31.3)

*Note:* Z refers to z-score difference between tests within each neuropsychological pearl (Pearl 1 = Letter-Category Fluency; Pearl 2 = HVLT-R Delay - WMS-III LM Delay; Pearl 3 = HVLT-R Delay – HVLT-R Recognition).

## Statement of Research Significance

### Research Question(s) or Topic(s):

- This study aimed to explore whether three cognitive patterns, or “clinical pearls,” of executive dysfunction could be confirmed in a large group of people with Parkinson’s disease (PD). The focus was three key cognitive pearls: letter < category fluency, delayed recall of a word list < story recall, and delayed recall < recognition.

### Main Findings:

- Statistically, the findings supported these patterns on average. However, significant variation was seen across individuals and the effect sizes were small for two pearls. This variability highlights the complexity of cognitive changes in PD.
